# Naïve, Regulatory, Activated, and Memory Immune Cells Co-exist in PVATs That Are Comparable in Density to Non-PVAT Fats in Health

**DOI:** 10.3389/fphys.2020.00058

**Published:** 2020-02-11

**Authors:** Ramya K. Kumar, Yining Jin, Stephanie W. Watts, Cheryl E. Rockwell

**Affiliations:** Department of Pharmacology and Toxicology, Michigan State University, East Lansing, MI, United States

**Keywords:** perivascular adipose tissue, immune cells, T cells, B cells, macrophages, NK cells, rats, flow cytometry

## Abstract

Perivascular adipose tissue (PVAT), the fat surrounding peripheral blood vessels, is protective and reduces the contraction of blood vessels in health. PVAT is composed of adipocytes, stromal cells, and immune cells. Recent work supports eosinophils as one of the cell types key to the anti-contractile nature of PVAT in health. Hence, we hypothesized that there exists a basally activated immune cell community in healthy PVAT that is distinctly different from non-PVAT fats. PVATs were from around mesenteric resistance vessels (MRPVAT – white fat) and thoracic aorta (APVAT – brown fat). Non-PVATs included retroperitoneal (RP fat – white fat) and subscapular (SS fat – brown fat) while the spleen was a positive control. Tissues were harvested from adult male and female Sprague Dawley rats. Six primary immune cell types were identified in PVATs. T cells (CD4 and CD8), B cells, natural killer (NK) cells, macrophages, mast cells, and neutrophils in the stromal vascular fraction of each fat were identified using nine-color flow cytometry. PVATs contained a higher number of total immune cells vs. their respective non-PVAT fats in females. Females had a higher number of T cells in MRPVAT vs. males. Females also had a greater number of T cells and total immune cells in APVAT vs. males. Further, activation, differentiation, and/or polarization of various immune cell types were similarly determined by flow cytometry. PVATs were similar to their respective non-PVAT fats in density of recently activated B cells (B220+ CD25+). However, MRPVAT in females had a higher number of naïve CD4 T cells vs. MRPVAT in males and APVAT in females. MRPVAT also had denser naïve CD8 T cells vs. APVAT in females. Overall, this research for the first time has identified a community of discrete populations of immune cells (naive/recently activated/regulatory/memory) in healthy PVATs. Contrary to our hypothesis, PVATs are more similar than different in density to their respective non-PVAT fats.

## Introduction

Perivascular adipose tissue (PVAT) contains heterogeneous cell populations including adipocytes, pre-adipocytes, fibroblasts, immune cells, endothelial cells, and nerves (Meijer et al., 2011; Szaz and Webb, 2012). Like many other organs, adipose tissues including PVATs are likely subject to immune surveillance. Type-2 immune cells [regulatory T cells (Tregs), invariant natural killer cells (iNKT), M2-like macrophages, and eosinophils] and their associated cytokines [interleukin (IL)-4, IL-5, IL-13, IL-10, and transforming growth factor-β] have been identified in healthy non-PVAT white and brown adipose tissues (Feuerer et al., 2009; Chawla et al., 2011; Nguyen Dinh Cat et al., 2011; Wu et al., 2011; Schipper et al., 2012a; Lynch et al., 2016). In health, the immune cells in non-PVAT fats interact with each other and also with other cell types in the adipose tissue, contributing toward maintaining the anti-inflammatory status of the tissue and preserving insulin sensitivity (Schipper et al., 2012b). They also contribute to brown fat activation, thermogenesis, white fat browning, and clearing cellular debris in healthy PVATs (Schipper et al., 2012b). B cells secrete immunoglobulin M antibodies that promote phagocytosis of apoptotic cells in healthy white adipose tissue (WAT) (Baumgarth, 2011). Although eosinophils, Tregs, and macrophages have been identified in healthy PVATs, only eosinophils in PVATs from healthy mice have been recognized as a key cell type to the anti-contractile nature of PVAT (Withers et al., 2017). Could there be a community of immune cells in PVAT that may be responsible for the anti-contractile nature of PVAT in health? This question can’t be answered until we know the relative immune composition and activation status of immune cells in PVATs in steady-state health. *Hence, our current study was focused on discovering the primary immune cell types and their basal activation status in PVATs, relative to the respective non-PVAT fats in both males and females in health.*

Perivascular adipose tissues are in close proximity to the blood vessels and may directly influence vascular tone, differing from non-PVAT fats (Gollasch, 2012). As such, we tested the hypothesis that PVAT contains a basally activated immune cell community in health, distinct from their respective non-PVAT fats. We postulate such an immune cell community to be protective in health. The current study focused on flow cytometric analyses of two PVATs – MRPVAT (WAT-like PVAT, located around small mesenteric resistance vessels) and APVAT (brown adipose tissue-like PVAT, located around thoracic aorta). Two non-PVAT adipose tissues – retroperitoneal fat (RP fat, non-PVAT white fat, found behind the kidneys) and subscapular fat (SS fat, non-PVAT brown fat, situated at the back of the neck region) – were used as non-PVAT fat-type comparators for MRPVAT and APVAT, respectively. Use of these four tissues from the same rats helped us answer two questions. First, are MRPVAT and APVAT different in immune composition? Second, are the PVATs different from their like non-PVAT fats? The spleen served as a positive control, given that it is a well-characterized secondary lymphoid organ. This also added confidence to the immunophenotyping data obtained in PVATs and other fats. This was especially important given the scarcity of flow cytometry work with rat adipose tissues, unlike mouse adipose tissues. We discovered a steady-state immune population in PVATs in health, a portion of which are basally activated, differentiated, and/or polarized. But in contrast to our hypothesis, the immune subpopulations of PVATs are more similar in density than different to their respective non-PVAT fats.

## Materials and Methods

### Animals

Animal maintenance and experimental protocols were approved by the Michigan State University Institutional Animal Care and Use Committee and complied with the National Institutes of Health Guide for Animal Care and Use of Laboratory Animals (2011). Male and female Sprague Dawley rats (350 g males and 250 g females, between 12 and 14 weeks of age, Charles River, Indianapolis, IN, United States; RRID: RGD_10395233) were used. Animals were maintained on a 12/12 light/dark cycle at 22–25°C. They were fed *ad libitum* (#8640 irradiated Teklad 22/5 rodent diet). Prior to all dissections, the rats were anesthetized with sodium pentobarbital (60–80 mg/kg, i.p.) and death was assured by creating a bilateral pneumothorax. Tissue dissection/processing proceeded as described below in the section “Immune Cell Isolation and Flow Cytometry.”

### Antibodies Used

Supplementary Tables 1, 2 list the antibodies used for immunophenotyping studies.

### Immune Cell Isolation and Flow Cytometry

Using flow cytometry, innate immune cells (macrophages, neutrophils, and mast cells), adaptive immune cells (T cells, B cells), and NK cells were identified and quantified. Live cells were either determined by using propidium iodide staining separately or Zombie aqua stain added to each cell preparation. Consistently, approximately 85–90% viable cells were obtained in every sample preparation. All the tissues were harvested from the same animals. Immune cells are reported as number of cells normalized to tissue weight in milligrams. The flow cytometric data were analyzed using Attune NxT software (v 2.6). An unstained control sample for each tissue type was used to: (i) adjust forward and side scatter so that the cell populations of interest are on scale and (ii) adjust the photomultiplier tube gain for each fluorochrome detector so that the peak mean fluorescence intensity of each channel was within 10^3^ and 10^4^ on a log scale. Spectral overlap was auto-compensated using single color compensation controls using compensation beads (Cat No. 01-2222-42) and the same compensation values were applied to all the tissues/rats.

### Splenocyte Isolation and Processing

Spleens were mechanically disrupted by a syringe plunger and filtered through a 40 μm filter. The single cell suspension obtained was then washed with Dulbecco’s modified eagle medium. Red blood cell lysis was performed by adding ammonium–chloride–potassium lysis buffer and incubating for 2 min on ice. The splenocytes were washed twice with PBS containing 1% fetal bovine serum (FACS buffer) and labeled with fluorescent antibodies after FcR blocking (CD32, Cat No. 550271). Viability was assessed with propidium iodide (1:30 in flow buffer, Cat No. 421301) immediately before analysis. In flow cytometry studies assessing steady state status of immune cells, viability was measured using Zombie-aqua dye (1:1000 in dPBS, 77143). All flow cytometry assays were performed using Attune NxT acoustic focusing cytometer from Life Technologies.

### Stromal Vascular Fraction Isolation From Adipose Tissues

APVAT, MRPVAT, RP fat, and SS fat were all dissected from the same rats. All the immune cell populations were quantified from the same fat samples, while another set of experiments were performed to phenotype the activation, differentiation, and/or polarization of various immune populations. The fats were removed from the blood vessels where appropriate, blotted dry, and weighed. The adipose tissues were minced with scissors, collagenase (1 mg/ml; type-I, Cat No. LS004196) digested at 37°C for about 1 h. The cell suspensions were sequentially filtered through 100 and 40 μm filters. The flow through contained cells lesser than 40 μm, so adipocytes were eliminated. Upon washing with flow buffer and centrifugation at 300 rcf for 5 min, a cell pellet which is called the stromal vascular fraction (SVF), was obtained.

### Surface Labeling of Immune Cells

Ammonium–chloride–potassium red blood cell lysis buffer (400 μl; Cat No. 10-548E) was added to the SVF pellet, gently pipette-mixed and incubated on ice for 2 min to destroy red blood cells. The red blood cell-lysed SVF was washed twice with FACS buffer and labeled with fluorescent antibodies (30 min incubation) after blocking Fc receptors with purified anti-CD32 antibody (10 min).

### Intracellular Labeling to Identify T Regulatory Cells

After the surface labeling was complete (described above), intracellular labeling was performed to identify cells containing Foxp3 as Tregs, using the Foxp3 transcription factor staining buffer set (Cat No. 5523). The cells were incubated with a fixation/permeabilization buffer for 1 h at room temperature in the dark and centrifuged at 700 rcf for 5 min. The supernatants were carefully discarded (pipetted out to minimize loss of cells) and the cells were washed with permeabilization buffer. The supernatants were carefully discarded again after centrifugation at 700 rcf. The intracellular label (Foxp3 for Treg cells) prepared in permeabilization buffer was incubated with the cells at room temperature for 30 min in the dark. The cells were then washed twice with permeabilization buffer and resuspended finally in FACS buffer for flow cytometry analysis.

### Defining Immune Cell Subtypes

CD4 T and CD8 T cells were further classified either as *naïve cells*, *recently activated cells* expressing early/late activation marker, *regulatory or memory cells*. B cells expressing CD25 were defined as *recently activated cells.* Macrophages were classified as either *M1-like or M2-like.* Supplementary Table 2 and Table 1 list the panel design details and specific definitions, respectively, for each of the identified subpopulations.

**TABLE 1:** Definitions of specific immune sub-populations.

**Surface markers**	**Definitions**	**References**
**T cell subtypes**		
CD4+Foxp3−CD25+ or CD8+Foxp3−CD25+	Recently activated T cells (early marker)	Stephens et al., 2004
CD4+OX40+ or CD8+OX40+	Recently activated T cells (late marker)	Stephens et al., 2004
CD4+Foxp3+ or CD8+Foxp3+	Regulatory T cells	Abe et al., 2009
CD4+CD25−CD45RC− or CD8+CD25−CD45RC−	Memory T cells	Luettig et al., 2001; Han et al., 2017
CD4+CD25−CD45RC+or CD8+CD25−CD45RC+	Naïve T cells	Luettig et al., 2001
**B cell subtype**		
B220+CD25+	Recently activated B cells	Amu et al., 2006
**Macrophage subtypes**		
CD68+CD86+MHCII+	Classically activated M1-like macrophages	Wang et al., 2017
CD68+CD163+	Alternatively activated M2-like macrophages	Yu et al., 2016

### Data Presentation and Statistics

Statistical analyses were performed with GraphPad Prism 8.0 (GraphPad Software Inc., La Jolla, CA, United States; RRID: SCR_002798). Gating for immune cells was done by three people (two blinded and one unblinded), analyses were done twice by one person to avoid any bias and ensure rigor and reproducibility. All gating and analyses yielded very similar results. Hence, data presented in this paper are from one person’s gating and analyses. Two-way ANOVA with Tukey’s multiple comparison test was used to determine statistical significance in all the flow cytometry experiments. Thus, two types of comparisons were possible: between the fats within each sex and between the sexes within each fat. A *P* < 0.05 was considered to be statistically significant.

Statistical power was calculated *a priori*. However, because it is very hard to define a specific effect size for biologically relevant differences in immune cell populations between adipose tissues, we had to use a “best guess approach” based on our preliminary data. Standard deviations for power analyses were obtained by averaging data (cell counts and percentages) on CD68+ macrophages and CD3+ T cells from a mix of males and females from preliminary studies done in the lab. The average standard deviation observed was 34% of the mean values. We chose a standardized effect size (Cohen’s *d*) of 0.8, as that is a standard criterion used to define a “large” effect size (i.e., biologically meaningful). The “*n*” value per group suggested for a Cohen’s *d* of 0.8, a power of 0.8 and 5% type-I error rate was 26. Hence, we acknowledge that our studies (*n* = 3–6), like most others in the field, are underpowered. However, since fractions of immune cells in adipose tissue have been reported to increase dramatically (e.g., ∼5% macrophages to ∼50% macrophages) during weight gain (Weisberg et al., 2003), we are confident that our analyses would be adequate to detect such large, biologically relevant changes as statistically significant.

## Results

### Gating Strategy to Identify and Characterize Immune Cells in PVATs

**FIGURE 1:**
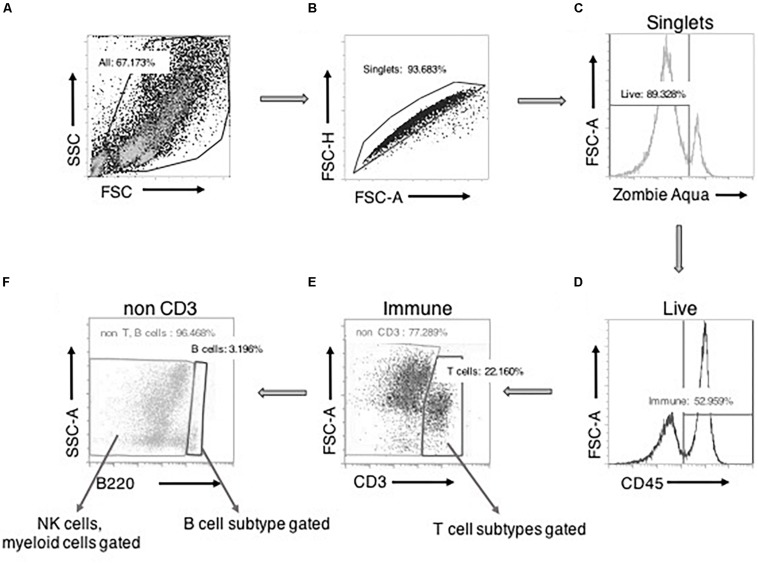
Basic gating strategy for flow cytometry experiments. A side-scatter (SSC) vs. forward scatter (FSC) plot with all the events recorded. Cells were selected “All” after size-based exclusion of cellular debris **(A)**. Forward scatter- height vs. area plot allowed for selection of “singlets,” avoiding cells sticking to each other as doublets **(B)**. “Viable/live” cells (zombie aqua negative) were then positively gated from the singlets **(C)**. From this live cell pool, CD45^+^ “immune” cells were gated **(D)**. From the immune cells, CD3^+^ “T cells” were selected. From this CD3+ cells, CD4/CD8 T cells and their subpopulations were further gated **(E)**. Then from the CD3^–^ cells, B220 expressing cells were gated as B cells. B220^+^CD25^+^ cells were further gated from B cells. CD3^–^B220^–^ cells were further gated into NK cells or myeloid cells and their subtypes **(F)**.

Figure 1 depicts our general strategy for identifying CD3+ T cells, B220+ B cells, and CD68+ macrophages along with their subpopulations, CD161+ NK cells, HIS48+ neutrophils, and FcεRI+ mast cells by flow cytometry. Cell clumps and cellular debris were first excluded by analysis of cell size on a forward-side scatter plot (Figure 1A). This step appreciably reduced autofluorescence in SVF preparations. Then, the doublets (Figure 1B) and Zombie aqua+ dead cells (Figure 1C) were excluded. Next, CD45+ leukocytes were selected (Figure 1D), followed by CD3+ T cell selection. CD3+ T cells were further classified into CD4+ and CD8+ T cells and their subpopulations (Figure 1E). From the CD3− cells, B220+ B cells (and CD25+ B cells) were selected (Figure 1F). Then, from the CD3−B220− population, individual innate immune cell niches that included macrophages (and their subtypes), NK cells, neutrophils, and mast cells were selected (Figure 1F) as shown by the markers in Supplementary Tables 1, 2.

### PVATs Contained a Higher Number of Immune Cells vs. Their Respective Non-PVAT Fats, in Female Rats Only

**FIGURE 2:**
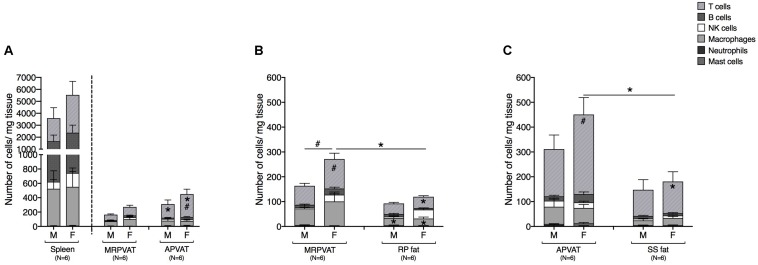
PVATs contained a denser immune cell community vs. their respective non-PVAT fats in female rats only. Immunophenotyping of mesenteric resistance (MR)PVAT, thoracic aortic (A)PVAT, retroperitoneal (RP) fat, subscapular (SS) fat, and spleen, in healthy male and female SD rats. T cells, B cells, natural killer (NK) cells, macrophages, neutrophils, and mast cells were quantified by flow cytometry. Each immune cell type is presented as absolute cell counts normalized to tissue weight (in mg). Comparisons between **(A)** MRPVAT and APVAT along with spleen. APVAT had a higher number of T cells (both sexes) per mg tissue vs. MRPVAT. Females had a greater number of T cells vs. males in APVAT **(B)** MRPVAT and non-PVAT white RP fat. MRPVAT had a higher number of macrophages (both sexes), T cells (females), and total immune cells (females) vs. RP fat. Females had a higher number of T cells and total immune cells in MRPVAT vs. males **(C)** APVAT and non-PVAT brown SS fat. APVAT had a higher number of T cells (females) and total immune cells (females) vs. SS fat. Females had a greater number of T cells in APVAT vs. males. Bars represent means ± SEM for the number of animals indicated by *N*. A *P* < 0.05 by two-way ANOVA was considered statistically significant. * and # inside the stacks in the graph represent a significant difference in the specific stack of immune cells between the two fats within each sex or between the sexes within each fat, respectively. * and # outside the stacks in the graph represent a significant difference in the sum total of immune cells between the two fats within each sex or between the sexes within each fat, respectively.

Six primary immune cell types: T cells, B cells, macrophages, NK cells, mast cells, and neutrophils were quantified by flow cytometry in PVATs. Spleen served as the positive control. APVAT had a higher number of T cells (both sexes) per mg tissue vs. MRPVAT and, the spleen contained 10 times greater number of immune cells vs. both the PVATs (both sexes) (Figure 2A). Females had a greater number of T cells vs. males in APVAT (Figure 2A). MRPVAT had a higher number of macrophages (both sexes), T cells (females), and total immune cells (females) vs. RP fat (Figure 2B). Females had a higher number of T cells and total immune cells in MRPVAT vs. males (Figure 2B). APVAT had a higher number of T cells (females) and total immune cells (females) vs. SS fat. Females had a greater number of T cells in APVAT vs. males (Figure 2C). Raw data values with average number of each immune cell type (per milligram tissue) in PVATs, non-PVAT fats, and spleen are presented in Table 2.

**TABLE 2:** Immune subpopulations (for data graphed in Figure 2) in spleen, MRPVAT, APVAT, RP fat, and SS fat in male and female SD rats.

	**Spleen**	**MRPVAT**	**APVAT**	**RP fat**	**SS fat**
**T cells**					
Male	1926.5 ± 877.5	76.2 ± 10.2	190 ± 57.1	42.7 ± 4.6	105.4 ± 41.1
Female	3162.3 ± 1130.3	118.8 ± 23.8	320.7 ± 68.3	45.8 ± 4.2	127.2 ± 39.5
**B cells**					
Male	1038 ± 516.4	10.4 ± 2.5	17.6 ± 4.5	10.1 ± 2.7	10.4 ± 2.7
Female	1628.4 ± 634.4	24.7 ± 5.6	33.1 ± 8.9	5.8 ± 0.7	9.7 ± 2.4
**NK cells**					
Male	97.2 ± 30.5	4.9 ± 0.96	24.3 ± 6.2	5.7 ± 3.4	7.4 ± 1.5
Female	193.1 ± 70.3	27.4 ± 10.9	22.6 ± 4.9	36.1 ± 6.9	10 ± 2.8
**Macrophages**					
Male	515.1 ± 250.4	66.6 ± 17.5	69.3 ± 34.9	29.5 ± 9.5	19.5 ± 5.7
Female	538.7 ± 215.2	98 ± 31.1	62.1 ± 14.4	29.1 ± 6.2	29.3 ± 6.3
**Neutrophils**					
Male	4.5 ± 3	1.9 ± 0.6	5.3 ± 2.1	1.8 ± 0.5	1.3 ± 0.4
Female	4.8 ± 1.9	1.1 ± 0.4	2.4 ± 1.1	0.5 ± 0.1	0.5 ± 0.2
**Mast cells**					
Male	4.9 ± 1.6	3.5 ± 1.4	4.6 ± 1.5	2.6 ± 1.5	3.4 ± 1
Female	7.2 ± 2.3	1.3 ± 0.7	9.8 ± 3.7	2.2 ± 1.1	4.1 ± 1.5

*Data presented in the table are average numbers of each immune cell type per milligram tissue ± SEM. Statistics are presented in Figure 2.*

### MRPVAT in Females Had a Higher Naïve CD4 T Cell Population vs. APVAT (Females) and MRPVAT (Males)

**FIGURE 3:**
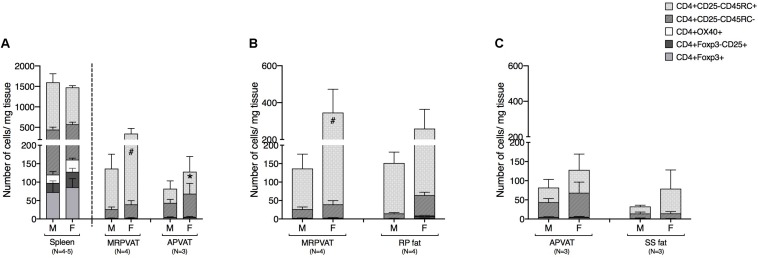
MRPVAT in females had a higher naïve CD4 T cell population vs. APVAT (females) and MRPVAT (males). Immunophenotyping of mesenteric resistance (MR)PVAT, thoracic aortic (A)PVAT, retroperitoneal (RP) fat, subscapular (SS) fat, and spleen, in healthy male and female SD rats. CD4 T cell subtypes (CD4+ CD25+, CD4+ OX40+, CD4+ Foxp3+, CD4+ CD45RC–, and CD4+ CD45RC+ cells) were quantified by flow cytometry. Each immune cell type is presented as absolute cell counts normalized to tissue weight (in mg). Comparisons between **(A)** MRPVAT and APVAT along with spleen. MRPVAT in females had a greater number of naïve CD4 T cells vs. APVAT in females and MRPVAT in males **(B)** MRPVAT and non-PVAT white RP fat. Females had a greater number of naïve CD4 T cells vs. males in MRPVAT **(C)** APVAT and non-PVAT brown SS fat. Bars represent means ± SEM for the number of animals indicated by *N*. A *P* < 0.05 by two-way ANOVA was considered statistically significant. * and # inside the stacks in the graph represent a significant difference in the specific stack of immune cells between the two fats within each sex or between the sexes within each fat, respectively.

CD4 T cells were classified as recently activated (expressing early marker CD25 or late marker OX40), regulatory (Foxp3+), memory (CD45RC−), or naïve (CD45RC+) in PVATs and their respective non-PVATs. MRPVAT in females had a greater number of naïve CD4 T cells vs. APVAT in females and MRPVAT in males (Figure 3A). The spleen contained approximately seven times greater number of total CD4 T cell subtypes vs. both the PVATs (both sexes) (Figure 3A). MRPVAT in females had a higher number of naïve CD4 T cells vs. males (Figure 3B). MRPVAT and APVAT had similar density of the CD4 T cell subpopulations that were analyzed vs. RP fat and SS fat, respectively (Figures 3B,C).

### MRPVAT Had a Greater Density of Naïve CD8 T Cells vs. APVAT in Female Rats Only

**FIGURE 4:**
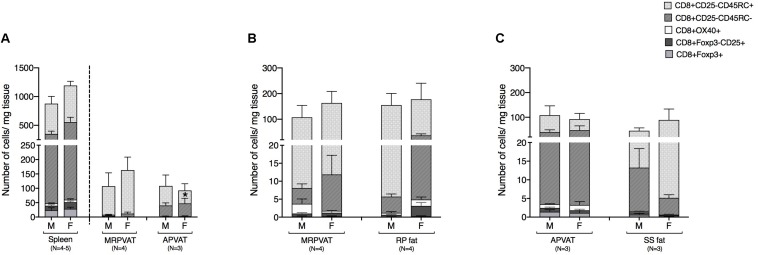
PVATs were mostly similar in numbers of CD8 T cell subtypes vs. their respective non-PVAT fats. Immunophenotyping of mesenteric resistance (MR)PVAT, thoracic aortic (A)PVAT, retroperitoneal (RP) fat, subscapular (SS) fat, and spleen, in healthy male and female SD rats. CD8 T cell subtypes (CD8+CD25+, CD8+OX40+, CD8+Foxp3+, CD8+CD45RC–, and CD8+CD45RC+ cells) were quantified by flow cytometry. Each immune cell type is presented as absolute cell counts normalized to tissue weight (in mg). Comparisons between **(A)** MRPVAT and APVAT along with spleen. MRPVAT had a greater density of naive CD8 T cells vs. APVAT in females **(B)** MRPVAT and non-PVAT white RP fat **(C)** APVAT and non-PVAT brown SS fat. Bars represent means ± SEM for the number of animals indicated by *N*. A *P* < 0.05 by two-way ANOVA was considered statistically significant. * inside the stack in the graph represents a significant difference in the specific stack of immune cells between the two fats within each sex.

CD8 T cell subpopulations were classified into naïve, recently activated, regulatory, and memory phenotypes, similar to that of CD4 T cells. MRPVAT had a greater density of naive CD8 T cells vs. APVAT in females only (Figure 4A). The spleen consisted of approximately seven times greater number of classified CD8 T cells vs. both PVATs (Figure 4A). MRPVAT and APVAT had similar density of the CD8 T cell subtypes that were analyzed vs. RP fat and SS fat, respectively (Figures 4B,C).

### PVATs Were Similar to Non-PVAT Fats in Density of CD25 Expressing B Cells

**FIGURE 5:**
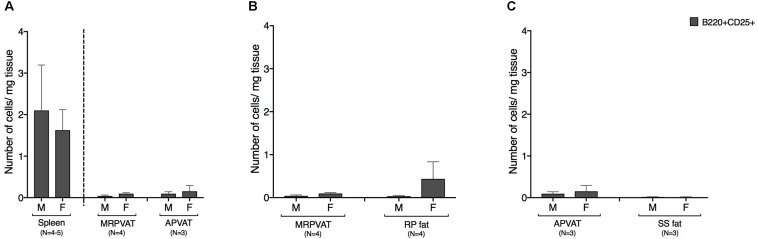
PVATs and non-PVAT fats have similar numbers of CD25 expressing B cells. Immunophenotyping of mesenteric resistance (MR)PVAT, thoracic aortic (A)PVAT, retroperitoneal (RP) fat, subscapular (SS) fat, and spleen, in healthy male and female SD rats. B220CD25 cells were quantified by flow cytometry and is presented as absolute cell counts normalized to tissue weight (in mg). Comparisons between **(A)** MRPVAT and APVAT along with spleen **(B)** MRPVAT and non-PVAT white RP fat **(C)** APVAT and non-PVAT brown SS fat. Bars represent means ± SEM for the number of animals indicated by *N*.

B cells expressing early marker CD25 were classified as recently activated. Both PVATs had a similar density of CD25+ B cells (Figure 5A). Spleen was composed of ∼10 times greater density of CD25 expressing B cells vs. both PVATs (Figure 5A). MRPVAT and APVAT had a similar CD25+ B cell density vs. RP fat and SS fat, respectively (Figures 5B,C).

### MRPVAT Contained an Increased Number of CD68+ Macrophage Subpopulations vs. APVAT and RP Fat, in Female Rats Only

**FIGURE 6:**
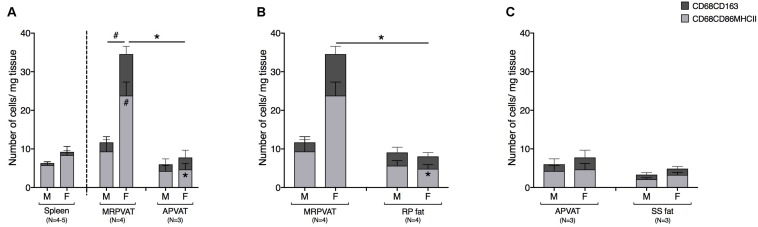
MRPVAT has a greater density of CD68+ macrophage subpopulation vs. APVAT and RP fat in females. Immunophenotyping of mesenteric resistance (MR)PVAT, thoracic aortic (A)PVAT, retroperitoneal (RP) fat, subscapular (SS) fat, and spleen, in healthy male and female SD rats. Mature CD68+ macrophages (CD68+CD163+ and CD68+CD86+MHCII+ cells) were quantified by flow cytometry. Each immune cell type is presented as absolute cell counts normalized to tissue weight (in mg). Comparisons between **(A)** MRPVAT and APVAT along with spleen. In females, MRPVAT had a greater number of CD68+CD86+MHCII+ macrophages and total CD68+ macrophage subpopulations vs. APVAT (females) and MRPVAT (males). **(B)** MRPVAT and non-PVAT white RP fat. In females, MRPVAT had a greater number of CD68+CD86+MHCII+ macrophages and total CD68+ macrophage subpopulations vs. RP fat **(C)** APVAT and non-PVAT brown SS fat. Bars represent means ± SEM for the number of animals indicated by *N*. A *P* < 0.05 by two-way ANOVA was considered statistically significant. * and # inside the stacks in the graph represent a significant difference in the specific stack of immune cells between the two fats within each sex or between the sexes within each fat, respectively. * and # outside the stacks in the graph represent a significant difference in the sum total of immune cells between the two fats within each sex or between the sexes within each fat, respectively.

The density of CD68+ macrophages in PVATs was comparable to that of spleen, save for MRPVAT in females that consist of approximately four times greater number of CD68+ macrophage subpopulations (Figure 6A). In females, MRPVAT had a greater number of CD68+ CD86+ MHCII+ macrophages and total CD68+ macrophage subpopulations vs. APVAT and male MRPVAT (Figure 6A) and vs. RP fat (Figure 6B). APVAT and SS fat had comparable numbers of CD68+ macrophage subtypes (Figure 6C).

### Immune Cell Subpopulations in Healthy PVATs Were More Similar Than Different vs. Non-PVAT Fats

**FIGURE 7:**
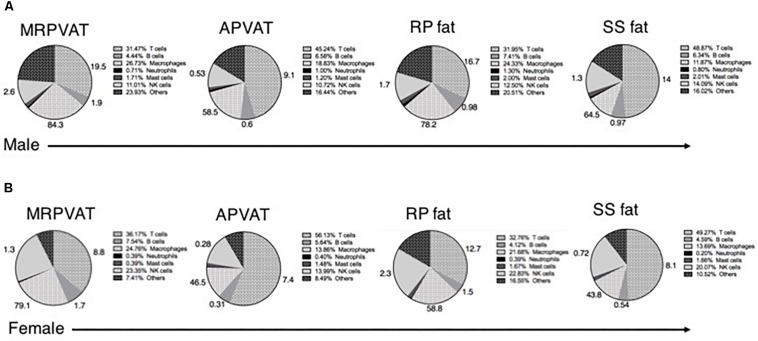
Summary. Pie chart representation of each immune cell type as a percentage of total immune composition in MRPVAT, APVAT, RP fat, and SS fat in males **(A)** and females **(B)**. The numbers outside represent the percentages of immune cell subpopulations listed in Table 1 (excluding naïve T cells) as percentage of each primary immune cell type in the respective pie sector.

Six primary immune cell types (both innate and adaptive) co- exist in PVATs and these can be subdivided based on activation, differentiation, and/or polarization status. Macrophages, T cells, B cells, and NK cells constituted about 80% of the total immune population in adipose tissues including PVATs (Figure 7). The numbers outside each wedge represent the percentages of the respective cell subpopulations that were classified [percentages of immune cell subpopulations listed in Table 1 (excluding naïve T cells), as percentage of each primary immune cell type in the respective pie sector]. This figure gives us an overview of the community of immune cells that are contained in PVATs. *PVAT vs. non-PVAT fat:* PVATs contained a similar percentage of each immune cell type vs. their respective non-PVAT fats. *White vs. brown fat:* MRPVAT and RP fat (white) were rich in CD68+ macrophages and CD68+ macrophage subtypes, while APVAT and SS fat (brown) were T-cell rich. *Males vs. Females:* Females (Figure 7A) consisted of a greater fraction of NK cells (MRPVAT, RP fat, and SS fat), T cells (APVAT), and a lesser percentage of “other” cells (all fats) defined as unidentifiable population of immune cells vs. males (Figure 7B). Raw data values with average percentages of each immune cell type in PVATs, non-PVATs, and spleen are presented in Table 3.

**TABLE 3:** Immune subpopulations (for data graphed as pie charts in Figure 7) in spleen, MRPVAT, APVAT, RP fat, and SS fat in male and female SD rats.

	**Spleen**	**MRPVAT**	**APVAT**	**RP fat**	**SS fat**
**T cells**					
Male	39.2 ± 4	31.2 ± 3.3	45.4 ± 6.8*	31.9 ± 4.1	48.6 ± 5.4
Female	40.9 ± 2.7	37.4 ± 4.8	56.7 ± 2.4*^#^	33.4 ± 2.1	50.4 ± 4.5
**B cells**					
Male	20 ± 2.8	4.4 ± 1	6.6 ± 2.4	7.4 ± 1.9	6.3 ± 1.1
Female	20.5 ± 2.1	7.8 ± 1.3	5.7 ± 1.2	4.2 ± 0.5	4.7 ± 1
**NK cells**					
Male	8.6 ± 0.6	10.9 ± 2.5	10.8 ± 1	12.5 ± 2.5	14.1 ± 2.8
Female	11.9 ± 2.3	24.1 ± 3.7	14.1 ± 3.1	23.3 ± 3	20.5 ± 2.7
**Macrophages**					
Male	10.5 ± 1.5	26.5 ± 7.5	18.9 ± 6.5	24.3 ± 7.2	11.8 ± 2.9
Female	7.6 ± 1	25.6 ± 6.8	14 ± 3.7	22.1 ± 4.8	14 ± 3.4
**Neutrophils**					
Male	0.1 ± 0.1	0.7 ± 0.2	1 ± 0.3	1.3 ± 0.4	0.8 ± 0.2
Female	0.1 ± 0	0.4 ± 0.1	0.4 ± 0.1	0.4 ± 0.1	0.2 ± 0.1
**Mast cells**					
Male	0.1 ± 0	1.7 ± 0.9	1.2 ± 0.2	2 ± 1.2	2 ± 0.4
Female	0.1 ± 0	0.4 ± 0.1	1.5 ± 0.3	1.7 ± 0.9	1.7 ± 0.3

Data presented in the table are average percentages of each immune cell type ± SEM. A *P* < 0.05 by two-way ANOVA was considered statistically significant. * represents a significant difference in T cell percentage between MRPVAT and APVAT (both sexes). # represents a significant difference in T cell percentage between males and females in APVAT.

## Discussion

The current study tested the hypothesis that an activated immune population exists in PVATs of healthy rats and that would be different vs. their respective non-PVAT fats. Flow cytometry was primarily employed. This work is important because: (1) the study describes a reliable method to isolate immune cells from multiple adipose tissues in rats and quantify them by flow cytometry; (2) it is the first time a study has been designed to compare the immune composition of two types of PVATs with their respective non-PVAT fat controls in both sexes in health; and (3) discrete immune subpopulations (naïve, recently activated, regulatory, and memory type) have been reported in PVATs in healthy rats for the first time.

Immune cell percentages/numbers identified in the spleen in the current study are consistent with the previous studies in Sprague Dawley rats (Morris and Komocsar, 1997). The current study has identified both innate and adaptive immune cells homing in healthy PVATs. Approximately 15–30% of immune cells were macrophages in adipose tissues including PVATs. This is similar to other reports where close to 15% of visceral adipose tissue is macrophages in lean mice, and are predominantly found in the interstitial spaces between adipocytes (Weisberg et al., 2003; Lumeng et al., 2008). In our present study, T, B, and NK cells accounted for nearly 40–50% of immune cells in healthy PVATs and non-PVATs. This is consistent with a recent report where lymphocytes (T and B cells) were ∼38% of live SVF cells in non-obese human abdominal subcutaneous WAT (Acosta et al., 2016). We observe a minor (∼2–3% of immune cells) mast cell and neutrophil population in PVATs and non-PVATs, which is also consistent with the existing literature in mice WAT (Ferrante, 2013). A distinct and substantial pool of memory T and B subsets have been identified in PVATs and non-PVATs here. This finding is also consistent with earlier mice studies that have revealed the presence of different subpopulations of memory T cells in non-PVAT WATs (Masopust et al., 2001; Han et al., 2017). By contrast, our study has identified ∼80–95% macrophages that are MHCII+ but other studies in mice that have reported 55% of MHCII+ macrophages in epididymal WAT in mice (Morris et al., 2013). This disparity in observations may be due to species differences in MHCII+ macrophages or due to different antibodies used for identifying macrophages (F4/80 in mice vs. CD68 in rats).

### In Health, Is the Immune System of PVATs Functionally Similar to Non-PVAT Fats?

The current study has identified a *similar* immune cell subtypes between PVATs and their respective non-PVAT fats in health. Our study has also revealed that MRPVAT and RP fat are rich in macrophages and T cells while APVAT and SS fat are T cell rich. Gene and protein expression studies from other labs reveal striking similarities between thoracic aortic PVAT and classical interscapular brown adipose tissue in mice (Fitzgibbons et al., 2011; Hildebrand et al., 2018) that are significantly different from WAT. This aligns well with our current findings and suggests that MRPVAT may be functionally similar to its respective non-PVAT RP fat, but different from APVAT. However, on the contrary, some previous studies also suggest that PVATs may be functionally different from their respective non-PVAT fats. Smooth muscle-specific peroxisome proliferator-activated receptor γ-knockout in mice resulted in selective loss of mesenteric and aortic PVAT, leaving the other fats (interscapular brown adipose tissue, gonadal WAT, and subcutaneous WAT) intact (Chang et al., 2012). This strongly supports that PVATs have different developmental properties than non-PVAT fats. Other studies have reported that visceral, gonadal, and subcutaneous fats hold distinct memory T cell gene signatures vs. spleen in mice (Han et al., 2017). This suggests that anatomically different adipose tissues may possess functionally distinct immune sub-populations. The close proximity of PVAT to the blood vessels (unlike other non-PVAT fats), the differences in the local tissue microenvironment and interaction with other immune/non-immune cells (Mahlakõiv et al., 2019) within the tissue are all likely to determine the fate and physiological functions of these immune cells in PVATs.

### Potential Roles of the Mature Immune Reservoir in PVATs

Finding what appears to be a recently activated, memory and/or polarized subtype of T cells, B cells, and macrophages in the PVATs of healthy rats raise a number of questions. *First*, why and how do immune cells in healthy PVATs become activated or acquire memory? *Second*, what functions do these different immune subtypes at steady state perform in healthy PVATs?

In our current study, the majority of T cells in adipose tissues including PVATs were either of naïve or memory phenotype. Non-PVAT WAT has an immune compartment that nurtures long-term maintenance in health and rapid re- activation of memory T cells in disease (Han et al., 2017). Over 80% of CD68+ macrophages are MHCII+ in PVATs in health. MHCII-mediated antigen presentation is critical for the development and maintenance of visceral adipose tissue Treg cells in healthy mice (Zeng et al., 2018). PVATs may serve similar functions and may participate in classical immunological host defense against pathogens. The endogenous activators of immune cells in PVATs in health have not yet been discovered. However, activated immune cells in PVATs may also be essential to produce regulatory molecules and other mediators to promote specific functions in other immune niches. WAT-resident anti-inflammatory macrophages, Tregs, and eosinophils directly/promote release of anti-inflammatory cytokines such as IL-10, transforming growth factor-β (Gong et al., 2012; Oh and Li, 2013; Grant and Dixit, 2015). These steady state immune cells and the cytokines released promote tissue repair and extracellular matrix remodeling, clear cell debris, aid in lipolysis, adipogenesis, angiogenesis, and overall help maintain insulin sensitivity (Schipper et al., 2012b). The paracrine action of PVAT on the vasculature may be much stronger than other adipose depots due to PVATs location relative to vascular tunica media and adventitia (Goodpaster et al., 2005; Gollasch, 2012; Noblet et al., 2015). Immune cells may either directly release factors that are anti-contractile or promote other cells in PVAT (e.g., adipocytes) to release relaxants. For example, IL-10 and IL-1β produced by macrophages and Tregs inhibit vascular smooth muscle cell contraction (Marceau et al., 2010; Zemse et al., 2010). PVAT-resident eosinophils directly release catecholamines that stimulate adipocytes to produce adiponectin and nitric oxide, via β-3 adrenoreceptors, which cause vasorelaxation (Withers et al., 2017).

*Third*, what provokes the protective PVAT-resident immune population to decline/maladapt in pathologies such as obesity, hypertension, and atherosclerosis? In disease, increased hypoxia/oxidative stress, excess free fatty acids, increased metabolic damage associated molecular patterns, and pattern associated molecular patterns are some known triggers that activate the innate immune cells which in-turn present the antigens to the adaptive immune cells in non-PVAT adipose tissues (Guzik et al., 2017). Further studies are warranted to identify the initiators and mechanisms of maladaptation of immune cells in PVAT in disease.

### Limitations

We recognize several limitations of our current work. First, the percentage of immune cells as determined by our gating strategy in each tissue does not total up to a hundred. Several reasons could explain this: (i) eosinophils and B cell subtypes could not be detected by flow cytometry due to lack of reliable surface markers/flow cytometry antibodies that can detect eosinophils and other B cell subtypes in rats; (ii) the adipose tissues could contain other immune cell types such as dendritic cells, type 2 innate lymphoid cells (ILC 2), and other immune cell types not expressing the flow markers that have been used in this study; (iii) PVATs and other fats of healthy rats contain unidentified/poorly defined immune cell populations. Second, we have not studied the contribution of immune cells in PVAT to arterial function. While important, this was beyond the scope of the present study which was dedicated to determine the immune cell community that exists in PVAT. Future studies to understand the functional capabilities (including arterial function) of the identified immune cells in PVAT are warranted. Third, the estrous staging of the female rats was not determined in this study. Fourth, lineage tracking studies would be necessary to clarify the origin of the immune cells identified in PVATs. Finally, collagenase digestion is the gold standard method for immune cell isolation from adipose tissues. Although the effect of collagenase on immune cell activation is unknown, interpretations should be made with caution as surface expression of markers could have been altered with enzymatic treatment.

## Conclusion

In summary, this study for the first time has identified discrete subpopulations of T cells, B cells, NK cells, and macrophages in healthy PVATs of both male and female Sprague Dawley rats, that are not distinctly different from non-PVAT fats. The current study not only highlights the similarities in the immune composition of PVATs vs. non-PVAT fats, males vs. females, but also the local heterogeneity of different PVATs in health. This leads to the question of what the different immune cell subtypes in healthy subtypes do and if we can exploit these similarities to develop broad-spectrum immunotherapeutic targets to white or brown fats including PVATs.

## Data Availability Statement

All datasets generated for this study are included in the article/Supplementary Material.

## Ethics Statement

The animal study was reviewed and approved by the Michigan State University Institutional Animal Care and Use Committee.

## Author Contributions

RK designed and performed the experiments, analyzed the data, and wrote the manuscript. YJ helped with the flow cytometry studies and revised the manuscript. SW and CR helped in designing the experiments, interpreted the data, and revised the manuscript.

## Conflict of Interest

The authors declare that the research was conducted in the absence of any commercial or financial relationships that could be construed as a potential conflict of interest.

## References

[B1] Abe Y. Urakami H. Ostanin D. Zibari G. Hayashida T. Kitagawa Y. (2009). Induction of Foxp3-expressing regulatory T-cells by donor blood transfusion is required for tolerance to rat liver allografts. *PLoS One* 4:e7840. 10.1371/journal.pone.0007840 19956764 PMC2776304

[B2] Acosta J. R. Douagi I. Andersson D. P. Bäckdahl J. Rydén M. Arner P. (2016). Increased fat cell size: a major phenotype of subcutaneous white adipose tissue in non-obese individuals with type 2 diabetes. *Diabetologia* 59 560–570. 10.1007/s00125-015-3810-6 26607638

[B3] Amu S. Gjertsson I. Tarkowski A. Brisslert M. (2006). B-cell CD25 expression in murine primary and secondary lymphoid tissue. *Scand. J. Immunol.* 64 482–492. 10.1111/j.1365-3083.2006.01832.x 17032240

[B4] Baumgarth N. (2011). The double life of a B-1 cell: self-reactivity selects for protective effector functions. *Nat. Rev. Immunol.* 11 34–46. 10.1038/nri2901 21151033

[B5] Chang L. Villacorta L. Li R. Hamblin M. Xu W. Dou C. (2012). Loss of perivascular adipose tissue on peroxisome proliferator-activated receptor- γ deletion in smooth muscle cells impairs intravascular thermoregulation and enhances atherosclerosis. *Circulation* 126 1067–1078. 10.1161/CIRCULATIONAHA.112.104489 22855570 PMC3493564

[B6] Chawla A. Nguyen K. D. Goh Y. P. S. (2011). Macrophage-mediated inflammation in metabolic disease. *Nat. Rev. Immunol.* 11 738–749. 10.1038/nri3071 21984069 PMC3383854

[B7] Ferrante A. W. (2013). The immune cells in adipose tissue. *Diabetes Obes. Metab.* 15(Suppl. 3), 34–38. 10.1111/dom.12154 24003919 PMC3777665

[B8] Feuerer M. Herrero L. Cipolletta D. Naaz A. Wong J. Nayer A. (2009). Lean, but not obese, fat is enriched for a unique population of regulatory T cells that affect metabolic parameters. *Nat. Med.* 15 930–939. 10.1038/nm.2002 19633656 PMC3115752

[B9] Fitzgibbons T. P. Kogan S. Aouadi M. Hendricks G. M. Straubhaar J. Czech M. P. (2011). Similarity of mouse perivascular and brown adipose tissues and their resistance to diet-induced inflammation. *Am. J. Physiol. Heart Circ. Physiol.* 301 H1425–H1437. 10.1152/ajpheart.00376.2011 21765057 PMC3197360

[B10] Gollasch M. (2012). Vasodilator signals from perivascular adipose tissue. *Br. J. Pharmacol.* 165 633–642. 10.1111/j.1476-5381.2011.01430.x 21486288 PMC3315036

[B11] Gong D. Shi W. Yi S. Chen H. Groffen J. Heisterkamp N. (2012). TGF-β signaling plays a critical role in promoting alternative macrophage activation. *BMC Immunol.* 13:31. 10.1186/1471-2172-13-31 22703233 PMC3406960

[B12] Goodpaster B. H. Krishnaswami S. Harris T. B. Katsiaras A. Kritchevsky S. B. Simonsick E. M. (2005). Obesity, regional body fat distribution, and the metabolic syndrome in older men and women. *Arch. Intern. Med.* 165 777–783. 10.1001/archinte.165.7.777 15824297

[B13] Grant R. W. Dixit V. D. (2015). Adipose tissue as an immunological organ. *Obesity* 23 512–518. 10.1002/oby.21003 25612251 PMC4340740

[B14] Guzik T. J. Skiba D. S. Touyz R. M. Harrison D. G. (2017). The role of infiltrating immune cells in dysfunctional adipose tissue. *Cardiovasc. Res.* 113 1009–1023. 10.1093/cvr/cvx108 28838042 PMC5852626

[B15] Han S.-J. Glatman Zaretsky A. Andrade-Oliveira V. Collins N. Dzutsev A. Shaik J. (2017). White adipose tissue is a reservoir for memory T cells and promotes protective memory responses to infection. *Immunity* 47 1154–1168.e6. 10.1016/j.immuni.2017.11.009 29221731 PMC5773068

[B16] Hildebrand S. Stümer J. Pfeifer A. (2018). PVAT and its relation to brown, beige, and white adipose tissue in development and function. *Front. Physiol.* 9:70. 10.3389/fphys.2018.00070 29467675 PMC5808192

[B17] Luettig B. Kaiser M. Bode U. Bell E. B. Sparshott S. M. Bette M. (2001). Naive and memory T cells migrate in comparable numbers through the normal rat lung: only effector T cells accumulate and proliferate in the lamina propria of the bronchi. *Am. J. Respir. Cell Mol. Biol.* 25 69–77. 10.1165/ajrcmb.25.1.4414 11472977

[B18] Lumeng C. N. DelProposto J. B. Westcott D. J. Saltiel A. R. (2008). Phenotypic switching of adipose tissue macrophages with obesity is generated by spatiotemporal differences in macrophage subtypes. *Diabetes* 57 3239–3246. 10.2337/db08-0872 18829989 PMC2584129

[B19] Lynch L. Hogan A. E. Duquette D. Lester C. Banks A. LeClair K. (2016). iNKT Cells induce FGF21 for thermogenesis and are required for maximal weight loss in GLP1 Therapy. *Cell Metab.* 24 510–519. 10.1016/j.cmet.2016.08.003 27593966 PMC5061124

[B20] Mahlakõiv T. Flamar A. L. Johnston L. K. Moriyama S. Putzel G. G. Bryce P. J. (2019). Stromal cells maintain immune cell homeostasis in adipose tissue via production of interleukin-33. *Sci. Immunol.* 4:eaax0416. 10.1126/sciimmunol.aax0416 31053655 PMC6766755

[B21] Marceau F. deBlois D. Petitclerc E. Levesque L. Drapeau G. Audet R. (2010). Vascular smooth muscle contractility assays for inflammatory and immunological mediators. *Int. Immunopharmacol.* 10 1344–1353. 10.1016/j.intimp.2010.08.016 20831918

[B22] Masopust D. Vezys V. Marzo A. L. Lefrançois L. (2001). Preferential localization of effector memory cells in nonlymphoid tissue. *Science* 291 2413–2417. 10.1126/science.1058867 11264538

[B23] Meijer R. I. Serne E. H. Smulders Y. M. van Hinsberg V. W. M. Yudkin J. S. Eringa E. C. (2011). Perivascular adipose tissue and its role in type 2 diabetes and cardiovascular disease. *Curr. Diab. Rep.* 11 211–217. 10.1007/s11892-011-0186-y 21461998 PMC3085790

[B24] Morris D. L. Cho K. W. Delproposto J. L. Oatmen K. E. Geletka L. M. Martinez-Santibanez G. (2013). Adipose tissue macrophages function as antigen-presenting cells and regulate adipose tissue CD4+ T cells in mice. *Diabetes* 62 2762–2772. 10.2337/db12-1404 23493569 PMC3717880

[B25] Morris D. L. Komocsar W. J. (1997). Immunophenotyping analysis of peripheral blood, splenic, and thymic lymphocytes in male and female rats. *J. Pharmacol. Toxicol. Methods* 37 37–46. 10.1016/s1056-8719(96)00146-3 9086287

[B26] Nguyen Dinh Cat A. Briones A. M. Callera G. E. Yogi A. He Y. Montezano A. C. (2011). Adipocyte-derived factors regulate vascular smooth muscle cells through mineralocorticoid and glucocorticoid receptors. *Hypertension* 58 479–488. 10.1161/HYPERTENSIONAHA.110.168872 21788604

[B27] Noblet J. N. Owen M. K. Goodwill A. G. Sassoon D. J. Tune J. D. (2015). Lean and obese coronary perivascular adipose tissue impairs vasodilation via differential inhibition of vascular smooth muscle K+ channels. *Arterioscler. Thromb. Vasc. Biol.* 35 1393–1400. 10.1161/ATVBAHA.115.305500 25838427 PMC4441615

[B28] Oh S. A. Li M. O. (2013). TGF-β: guardian of T cell function. *J. Immunol.* 191 3973–3979. 10.4049/jimmunol.1301843 24098055 PMC3856438

[B29] Schipper H. S. Prakken B. Kalkhoven E. Boes M. (2012a). Adipose tissue-resident immune cells: key players in immunometabolism. *Trends Endocrinol. Metab.* 23 407–415. 10.1016/j.tem.2012.05.011 22795937

[B30] Schipper H. S. Rakhshandehroo M. van de Graaf S. F. J. Venken K. Koppen A. Stienstra R. (2012b). Natural killer T cells in adipose tissue prevent insulin resistance. *J. Clin. Invest.* 122 3343–3354. 10.1172/JCI62739 22863618 PMC3428087

[B31] Stephens L. A. Barclay A. N. Mason D. (2004). Phenotypic characterization of regulatory CD4+CD25+ T cells in rats. *Int. Immunol.* 16 365–375. 10.1093/intimm/dxh033 14734622

[B32] Szaz T. Webb R. C. (2012). Perivascular adipose tissue: more than just structural support. *Clin. Sci.* 122 1–12. 10.1042/CS20110151 21910690 PMC3966487

[B33] Wang M. Fijak M. Hossain H. Markmann M. Nüsing R. M. Lochnit G. (2017). Characterization of the micro-environment of the testis that shapes the phenotype and function of testicular macrophages. *J. Immunol.* 198 4327–4340. 10.4049/jimmunol.1700162 28461571

[B34] Weisberg S. P. McCann D. Desai M. Rosenbaum M. Leibel R. L. Ferrante A. W. (2003). Obesity is associated with macrophage accumulation in adipose tissue. *J. Clin. Invest.* 112 1796–1808. 10.1172/JCI19246 14679176 PMC296995

[B35] Withers S. B. Forman R. Meza-Perez S. Sorobetea D. Sitnik K. Hopwood T. (2017). Eosinophils are key regulators of perivascular adipose tissue and vascular functionality. *Sci. Rep.* 7:44571. 10.1038/srep44571 28303919 PMC5356000

[B36] Wu D. Molofsky A. B. Liang H.-E. Ricardo-Gonzalez R. R. Jouihan H. A. Bando J. K. (2011). Eosinophils sustain adipose alternatively activated macrophages associated with glucose homeostasis. *Science* 332 243–247. 10.1126/science.1201475 21436399 PMC3144160

[B37] Yu E. Goto M. Ueta H. Kitazawa Y. Sawanobori Y. Kariya T. (2016). Expression of area-specific M2-macrophage phenotype by recruited rat monocytes in duct-ligation pancreatitis. *Histochem. Cell Biol.* 145 659–673. 10.1007/s00418-016-1406-y 26860866 PMC4848343

[B38] Zemse S. M. Chiao C. W. Hilgers R. H. P. Webb R. C. (2010). Interleukin-10 inhibits the in vivo and in vitro adverse effects of TNF-alpha on the endothelium of murine aorta. *Am. J. Physiol. Heart Circ. Physiol.* 299 H1160–H1167. 10.1152/ajpheart.00763.2009 20639218 PMC2957353

[B39] Zeng Q. Sun X. Xiao L. Xie Z. Bettini M. Deng T. (2018). A unique population: adipose-resident regulatory T cells. *Front. Immunol.* 9:2075. 10.3389/fimmu.2018.02075 30323806 PMC6172295

