# Supplementary material for: Naïve, Regulatory, Activated, and Memory Immune Cells Co-exist in PVATs That Are Comparable in Density to Non-PVAT Fats in Health
Source: Front Physiol. 2020 Feb 11;11:58. doi: 10.3389/fphys.2020.00058 (PMC7026504; doi:10.3389/fphys.2020.00058)
Supplement: Supplementary file 1 [file Table_1.DOCX]

**Supplementary Table-1** List of immunophenotyping antibodies

| Antibody | Fluorochrome | Supplier | Clone | Dilution |
| --- | --- | --- | --- | --- |
| Live/dead | Zombie aqua | Biolegend |  | 1:1000 |
| CD45 | APC/Cy7 | BD Bioscience | OX-1 | 1:200 |
| CD3 | PE | BD Bioscience | 1F4 | 1:100 |
| CD4 | BV605 | BD Bioscience | OX-35 | 1:50 |
| CD8a | PerCPeFluor710 | eBioscience | OX-8 | 1:100 |
| B220 | PE/Cy7 | BD Bioscience | OX-39 | 1:50 |
| CD68 | AF647 | Biorad | ED1 | 1:10 |
| CD161 | APC | Biolegend | 3.2.3 | 1:100 |
| HIS48 | FITC | BD Bioscience | HIS-48 | 1:100 |
| FcεRI | FITC | EMD Millipore | γ-subunit | 1:25 |

**Supplementary Table-2** List of antibody panels

| Antibody | Fluorochrome | Supplier | Clone | Dilution |
| --- | --- | --- | --- | --- |
| *Baseline* |  |  |  |  |
| Live/dead | Zombie aqua | Biolegend |  | 1:1000 |
| CD45 | APC/Cy7 | BD Bioscience | OX-1 | 1:200 |
| CD3 | FITC | BD Bioscience | 1F4 | 1:100 |
| *T cell panel* |  |  |  |  |
| CD4 | BV605 | BD Bioscience | OX-35 | 1:50 |
| CD8 | PerCPeFluor710 | eBioscience | OX-8 | 1:100 |
| CD25 | PE | BD Bioscience | OX-39 | 1:100 |
| CD134 | BV711 | BD Bioscience | OX-40 | 1:50 |
| CD45RC | AF680 | Santa Cruz | OX-22 | 1:50 |
| Foxp3 | AF647 | Biolegend | 150D | 1:20 |
| *B cell panel* |  |  |  |  |
| B220 | PE/Cy7 | eBioscience | HIS24 | 1:50 |
| CD25 | BV605 | BD Bioscience | OX-39 | 1:50 |
| *Macrophage panel* |  |  |  |  |
| CD68 | AF700 | Biorad | ED1 | 1:10 |
| CD86 | AF647 | Biolegend | 24F | 1:50 |
| CD163 | PE | Thermofisher | ED2 | 1:50 |
| MHCII | BV711 | BD Bioscience | OX-6 | 1:50 |
